# Cecal bascule herniation through the foramen of Winslow

**DOI:** 10.4322/acr.2020.236

**Published:** 2021-01-28

**Authors:** Aaron M. Williams, Zachary Pickell, Mary R. Shen, Naveen Sangji

**Affiliations:** 1 University of Michigan, Michigan Medicine, Department of Surgery, Ann Arbor, MI, USA

**Keywords:** Laparotomy, Colorectal Surgery, Intestinal Obstruction, Hernia

## Abstract

Bowel obstructions can have a variety of causes, including impacted feces, adhesions, volvulus, non-internal hernias, and in rare cases internal hernias. We report a 63-year-old woman who presented to the emergency department with severe abdominal pain, nausea, vomiting, and obstructive symptoms that had started 12 hours earlier. A computed tomographic scan of the abdomen and pelvis showed a right internal hernia with a cecal bascule traversing through the foramen of Winslow, concerning for a closed-loop obstruction. The patient underwent an exploratory laparotomy with cecal bascule reduction and cecopexy. Given the increased mortality risk if undiagnosed, it is important to remain aware of internal hernias. Patient outcomes are markedly improved with early diagnosis and surgical intervention.

## INTRODUCTION

A cecal bascule is a rare condition in which the cecum folds anteriorly and superiorly over the ascending colon.^1^ Although difficult to distinguish from a cecal volvulus, it typically presents with less acuity due to the absence of torsion of the mesenteric vasculature. A cecal bascule, however, most commonly presents with symptoms related to bowel obstruction. Importantly, these patients can present with a closed-loop obstruction in the presence of a competent ileocecal valve or rarely, in the setting of an internal hernia, as in this case. Diagnosis is often difficult due to its rare nature and often equivocal imaging findings. Once diagnosed, emergent surgical management is warranted including cecal bascule reduction followed by right hemicolectomy versus cecopexy based on colonic viability.^2^

## CASE REPORT

A 63-year-old woman with a history of gastroesophageal reflux disease (GERD) associated with a small hiatal hernia presented to the emergency department with severe abdominal pain, nausea, vomiting, and obstructive symptoms that had started 12 hours earlier. The pain was epigastric in nature. She reported no passing of flatus or bowel movement for 24 hours prior to presentation. She also reported a history of intermittent abdominal pain and obstipation that would self-resolve without treatment over the last several years. She had no prior surgical history. Her body mass index was 31. She denied any fever, chills, chest pain, or shortness of breath. Her vitals on presentation included a blood pressure of 110/70 mm Hg, heart rate of 105 beats per minute, and respiratory rate of 22 breathes per minutes. On physical examination, the abdomen was distended with focal tenderness in the epigastric and peri-umbilical regions. No peritoneal signs were noted.

Laboratory analysis revealed a leukocytosis to 12.9 K/uL (reference range [RR]; 4.0-10.0K/uL) with an elevation in creatinine to 0.8 mg/dL from 0.4 mg/dL (RR; 0.3 -0.6mg/dL). Her blood urea nitrogen level was elevated to 20 mg/dL from 8 mg/dL (RR; 4 – 8 mg/dL). Her lipase, amylase, and liver function tests were within normal limits. Her lactate level was mildly elevated to 3.0 mg/dL (RR; 0.5-1mmol/L).

**Figure 1:**
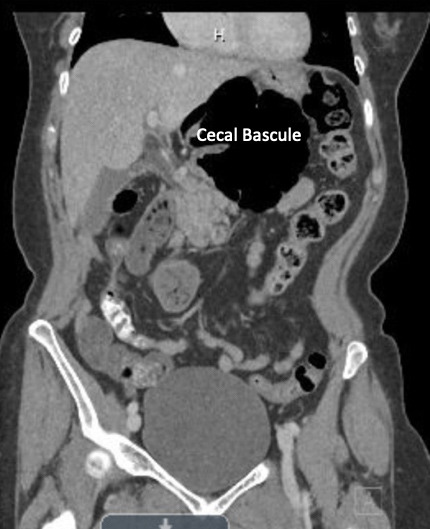
Computed tomographic scan of the abdomen and pelvis revealing cecal bascule herniation through the Foramen of Winslow.

A computed tomography (CT) scan of her abdomen and pelvis revealed a right internal hernia containing cecal bascule herniation through the foramen of Winslow into the lesser sac, concerning for a closed-loop obstruction (Figure 1). There was fecalization with upstream bowel dilation. There was no evidence of pneumoperitoneum, pneumatosis, or bowel wall compromise. A normal appendix was also noted in the right upper quadrant near the gallbladder.

The patient’s symptoms including pain, nausea, vomiting, no flatus or bowel movements, along with her leukocytosis and imaging findings were concerning for an internal hernia with a cecal bascule traversing the foramen of Winslow causing a closed-loop obstruction. The patient was taken to the operating room.

**Figure 2:**
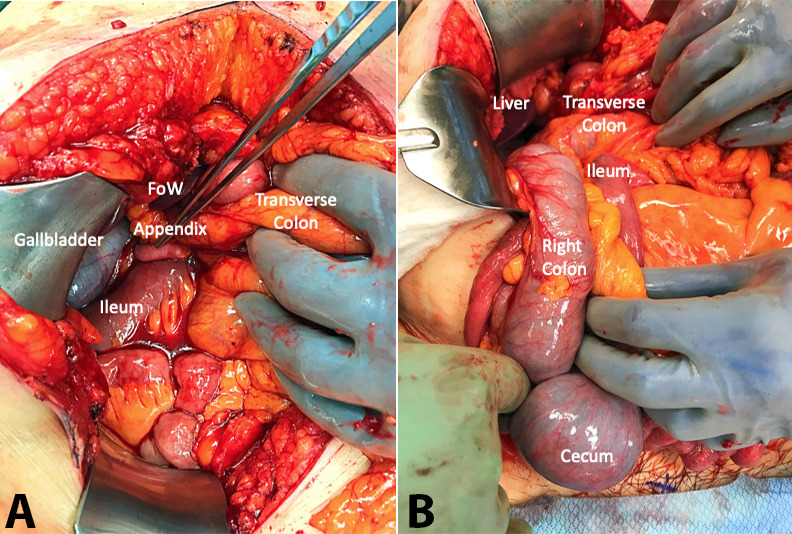
**A –** Intraoperative findings highlighting cecal herniation through the Foramen of Winslow. The appendix is noted in the right upper quadrant near the gallbladder. The ileum and transverse colon are shown traversing the foramen of Winslow. There is no evidence of the cecum; **B –** Intraoperative findings post-reduction of cecal bascule herniation through Foramen of Winslow. The cecum and right colon appeared viable.

She underwent an exploratory laparotomy. After fixed retractors were placed, the appendix was noted to be in the right upper quadrant near the gallbladder and liver edge (Figure 2A). The terminal ileum and transverse colon were noted to be tracking behind the portal triad. Upon closer examination, these structures were noted to be involved in an internal hernia with a cecal bascule herniating through the foramen of Winslow. Adhesions were lysed and the hernia sac was partially excised facilitating reduction of the cecal bascule (Figure 2B). The colon was noted to be viable. After reduction, the hernia sac was fully excised. The right colon mesentery was noted to be highly redundant (Figure 2B).

Given the viability of the cecum and right colon, the decision was made to perform a cecopexy rather than right hemicolectomy to minimize the risk of a recurrent internal hernia. An appendectomy was also performed. Omentum was placed near the foramen of Winslow to also help prevent future risk of a recurrent internal hernia. No attempt was made at suture closure of the foramen of Winslow space. The abdomen was closed in the standard fashion.

The patient’s postoperative course was uncomplicated. Her pain was well-controlled with oral pain medication. Her diet was advanced in a stepwise fashion. She was able to ambulate independently and void spontaneously upon discharge. The patient was discharged 4 days following her operation. She has also been doing well more than 6 months out from surgery.

## DISCUSSION

An internal hernia is a rare cause of intestinal obstruction, accounting for less than 5% of cases.^2^ Although rare, early diagnosis is crucial as an internal hernia can be associated with a high mortality rate secondary to bowel ischemia. The most common internal hernias can include paraduodenal, transmesenteric, paracecal, intersigmoid, paravesicle, and through the foramen of Winslow.^3^ Of these several types of internal hernia, nearly 8% involve herniation through the foramen of Winslow, which is the communication through the greater sac to the lesser sac behind the portal triad.^4^^,^^5^ Predisposing factors leading to internal herniation through the foramen of Winslow include an enlarged foramen of Winslow, failed retroperitonealization of the right colon, and a redundant intestinal mesentery.^6^

Although numerous organs have been known to herniate through the foramen of Winslow, cecal herniation is rare. Cecal herniation through the foramen of Winslow has only been reported in a limited number of cases. These cases include a cecal bascule herniation, cecal herniation along with the ascending colon and terminal ileum, cecal herniation with volvulus, and cecal herniation in the absence of a bascule.^6^^-^^9^ To our knowledge, there are close to 20 reports of cecal herniation through foramen of Winslow (Table 1).

**Table 1:** Cecal herniation through the foramen of Winslow

Year Reported	Age	Sex	Imaging Modality	Surgical Intervention
1973^10^	44	F	Abdominal plain films	Laparotomy with an appendicectomy
1974^11^	41	F	Abdominal plain films	Reduction of hernia with cecopexy
1974^11^	56	F	Abdominal plain films	Reduction of hernia with cecopexy
1977^12^	8	F	Abdominal plain films/barium enema	Laparotomy and partial resection of the ileum
1982^13^	46	F	Abdominal plain films/ultrasound	Laparotomy with an appendicectomy
1989^14^	84	F	Abdominal plain films/barium enema	Laparotomy with a right hemicolectomy
1991^15^	32	F	CT scan	Laparotomy with cecostomy, cecopexy, and an appendectomy
2001^16^	51	F	Abdominal plain films	Laparotomy with a right hemicolectomy
2009^17^	60	F	CT scan	Laparoscopic hernia reduction
2010^18^	45	F	Abdominal plain films/CT scan	Laparotomy with a right hemicolectomy
2010^19^	49	F	Abdominal plain films/CT scan	Laparotomy with a right hemicolectomy
2013^10^	62	M	CT scan	Laparotomy with a right hemicolectomy
2014^7^	75	F	CT scan	Laparotomy
2016^20^	38	F	CT scan	Laparotomy with an appendicectomy
2016^21^	57	M	CT scan	Laparotomy with a right hemicolectomy
2019^6^	56	F	CT scan	Laparotomy with a right hemicolectomy
2019^22^	80	F	CT scan	Laparotomy with a right hemicolectomy
2019^8^	66	F	Abdominal plain films/CT scan	Laparotomy with a right hemicolectomy
2020^11^	69	M	CT scan	Laparotomy

CT = computed tomography; F = Female; M = Male.

With the advent of computed tomography (CT) imaging, diagnosing cecal herniation through the foramen of Winslow preoperatively has become easier compared to abdominal plain films and barium enemas. A CT scan may help provide the best anatomical delineation and is now the standard method of detection.^2^ Interestingly, several case reports highlight cecal herniation through the foramen of Winslow following laparoscopic Nissen fundoplication.^16^^,^^23^ Given this association, further study is warranted. Presently, there is no consensus about the best surgical interventions to manage cecal herniation through the foramen of Winslow because of this rare internal hernia.^2^^,^^5^ Notably, despite differing surgical approaches, no published cases reported a re-occurrence of cecal herniation through the foramen of Winslow after surgical intervention.

**Figure 3:**
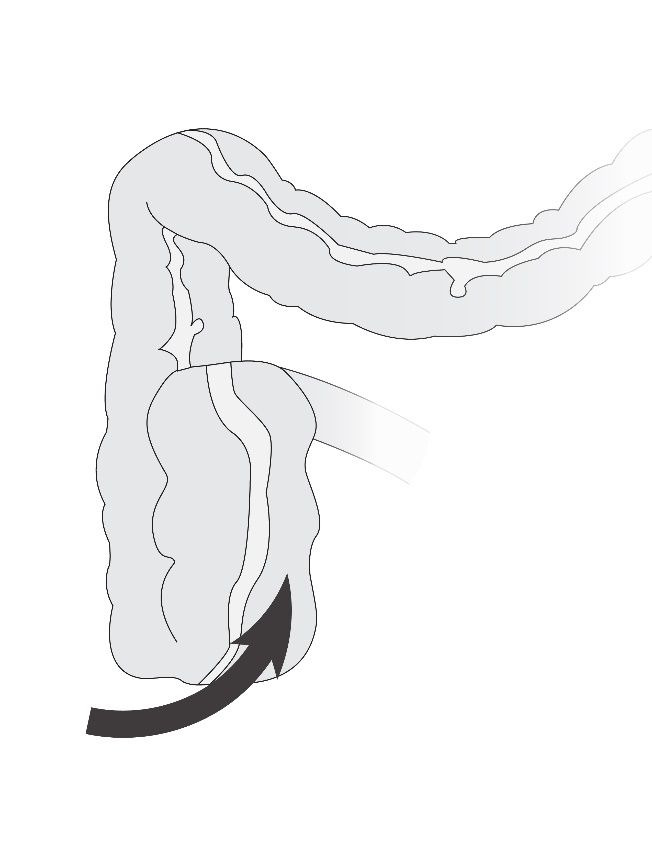
Cecal bascule: a rare subtype of internal hernia.

A cecal bascule is a rare condition where the hypermobile cecum can fold anteriorly and superiorly leading to obstruction (Figure 3). Differentiating a cecal bascule is difficult, as nearly 10-15% of cecal volvulus diagnoses turn out to be cecal bascules upon intraoperative findings.^24^ Hypermobility of the cecum along with marked bowel distention are well-known risk factors for development.^24^ Clinical presentation commonly includes obstructive symptoms including abdominal pain, nausea, vomiting, and decreased frequency of flatus and bowel movements. In this case, the CT scan revealed an internal hernia containing a cecal bascule traversing through the foramen of Winslow. To our knowledge, there are a limited number of reports in the literature describing a cecal bascule presenting as an internal hernia through the foramen of Winslow.^5^^,^^7^^,^^10^ In one report, however, the patient had prior pelvic surgery with possible cecal mobilization.^5^^,^^7^^,^^10^ Our patient, however, had a virgin abdomen with no prior surgical history with severe redundancy of the right colon mesentery leading to her presentation.

After a diagnosis of a cecal bascule herniation through the foramen of Winslow has been made, prompt definitive treatment is required to minimize the risk of strangulation. Although some reports describe laparoscopic approaches for internal hernias through the foramen of Winslow,^25^ an open laparotomy approach is most commonly needed, as was performed in this study. Our institutional approach is similar to other studies.^2^^,^^3^^,^^5^ Once adequate exposure is obtained, the goal should be to reduce the hernia contents using dissection and gentle retraction. If the foramen of Winslow is tight, this can be enlarged by using a Kocher maneuver. After reduction of the hernia, the bowel must be carefully inspected for viability. If the bowel is viable, a cecopexy may be performed, as was done in this case. However, if the bowel is determined to be compromised, a right hemicolectomy is required. Although a right hemicolectomy has been advocated by some to minimize recurrence even in the setting of viable bowel,^16^ there are no reported cases of re-herniation following cecopexy alone.^7^ Although controversial, some have also suggested to close the foramen of Winslow to minimize the risk of recurrence; however, any attempts may risk damage to the biliary ducts, hepatic artery, or portal vein leading to thrombosis or injury.^26^ Omentum, however, can be placed nearby to minimize risk of re-herniation as was performed in this case.^13^

## CONCLUSION

Patients who present with acute onset abdominal pain with associated gastrointestinal obstructive symptoms require prompt workup for bowel obstruction secondary to internal hernia or closed-loop obstruction. A cecal bascule through the foramen of Winslow with closed-loop obstruction is a rare condition that warrants emergent surgical management given risk of colon strangulation. The risks and benefits of cecopexy compared to a right hemi-colectomy must be weighed carefully based on intraoperative bowel viability.
